# Dermal measurement of exposure to plant protection products: Actual hand exposure from hand washing vs. wearing cotton gloves

**DOI:** 10.3389/fpubh.2022.1037780

**Published:** 2022-12-15

**Authors:** Christian J. Kuster, Nicola J. Hewitt, Georg Hamacher

**Affiliations:** ^1^Bayer AG, Crop Science Division, Monheim, Germany; ^2^Scientific Writing Services (SWS), Erzhausen, Germany; ^3^AGREXIS AG, Basel, Switzerland

**Keywords:** dermal exposure, hand wash, cotton gloves, dosimetry, plant protection products

## Abstract

For the authorization of plant protection products, a quantitative non-dietary exposure risk assessment relies on established dermal exposure models, measured mainly using passive dosimetry. Exposure to the hands is determined *via* hand washing or using cotton gloves as a surrogate for skin. This study compared both methods using operator exposure data available from the Agricultural Operator Exposure Model (AOEM) project report. These data indicate that hand exposure determined using cotton gloves resulted in markedly higher exposure values for all exposure scenarios compared to those determined by hand washes. One explanation for this is that dermal uptake of the residues reduces the amount of residue that can be recovered by hand washing. Uncertainty due to dermal uptake can be addressed by either default assumptions or by specific dermal absorption data. However, this cannot solely account for the large difference observed between the values and is mainly likely to be due to the higher capacity of the cotton gloves vs. human skin to retain residues. The results further indicate that the variability between hand wash samples and cotton glove samples differs between the exposure scenarios. Hence, the level of conservatism related to the use of cotton gloves as surrogate skin remains unknown. In conclusion, this evaluation of the AOEM data indicates that the cotton glove method results in much higher levels of measured hand exposure than the hand wash method. It cannot be excluded that dermal uptake has contributed to that result. However, the findings suggest the higher retention capacity of cotton gloves vs. human skin to be the main impact parameter. The cotton glove method does not provide the results with regards to the protection level that can be expected from the use of protective gloves. Therefore, we believe that the application of the hand wash method is a more accurate measure of exposure levels, if either specific dermal absorption data or, in its absence, default assumptions are applied as adjustment factor.

## 1. Introduction

The authorization of plant protection products (PPPs) used in agriculture routinely requires a quantitative non-dietary exposure risk assessment. This risk assessment relies on measures of exposure including operator exposure during mixing, loading, or the application of the PPP or through re-entry into a recently treated field. The Organization for Economic Co-operation and Development (OECD) and the European Food Safety Authority (EFSA) provide guidance on the design of studies to determine exposure to pesticides in agricultural settings, and how this information can be used in a tiered approach for risk assessment (1, 2). The correct determination of the exposure is critical to ensure a reliable and realistic risk assessment to determine if use of a PPP is considered safe for humans.

The determination of hand exposure in operator risk assessments is especially important, since hand exposure accounts for a significant portion of the overall exposure (3, 4). Methods for determining actual hand exposure include the use of dosimeters, removal techniques (e.g. hand washes), interception techniques (e.g. cotton gloves, patches, or coveralls) and fluorescent tracer techniques to provide a measure of the integrated exposure loading over the exposure duration (4–8). There are advantages and disadvantages to both methods, which is why the OECD guideline does not recommend a specific method. For example, with removal techniques (hand washed), dermal uptake can potentially impact the removal efficiency resulting in an underestimation of dermal exposure. For the interception techniques (cotton gloves), the adsorption and absorption capacities of the interception material might differ from skin properties. According to the OECD test guideline (2), “The US EPA (1987) stated that the use of gloves as a monitoring method may result in a significant overestimation of total dermal exposure, owing to their capacity to retain more of the pesticide than would be retained by the skin”.

To address the lack of standardization of the guidelines for measuring dermal exposure, the Federal Institute for Occupational Safety and Health (BAuA) conducted a “systematic analysis of dermal exposure to hazardous chemical agents at the workplace” (SysDEA) which was published in a report in 2020 (5). The study compared the advantages and disadvantages of three different methods for determining exposure and investigated ways to decrease the uncertainty in the measurements. The three methods included (1) the interception of chemicals (whole body dosimetry using coveralls, gloves, patches), (2) the removal by wiping or washing, and (3) *in situ* methods by fluorescence measurements. The experiments were carried out under standardized conditions in test chambers, following detailed protocols to increase reproducibility and reduce variability. Exposure to test substances were measured for different parts of the body (hands, body, head) during various activities e.g., mixing and loading of the concentrated product in the spray tank, and application of the diluted product with a handheld device or with a broadcast sprayer. The study concluded that cotton gloves were advantageous simply because they derived the most conservative measurement of hand exposure compared to the use of hand washing. However, the question remains if cotton gloves provide a realistic model to estimate non-dietary exposure. To address this, we conducted a comparative analysis of actual hand exposure data available from the Agricultural Operator Exposure Model (AOEM) project report. The AOEM project summarizes operator exposure studies measuring exposure toward PPPs following preparation (mixing and loading) and application using tractor mounted spray equipment (3). Thirty operator exposure studies conducted by agricultural industry mainly for the purpose of plant protection product authorization between 1994 and 2009 were evaluated to develop a generic operator exposure model, which represents current application techniques and practices in Europe. The new model (AOEM) is part of the EFSA calculator and routinely used for national authorization and registration procedures of plant protection products. Detailed information on the model development are described in the AOEM project report (9). In the AOEM project, actual hand exposure was either measured by sampling cotton gloves (analogous to method (1) in the SysDEA experiment) or by direct hand washes [SysDEA: Method (2)]. While other methods for exposure exist, none were evaluated in this study.

## 2. Material and methods

Actual hand exposure was evaluated using data taken from the AOEM project report (3). Exposure toward PPPs was measured following preparation (mixing and loading) and application of the spray using mechanical-assisted spray equipment.

If hand washes were used to determine actual hand exposure, hands of the operator were washed and thoroughly rinsed with a water-solvent mixture. The rinsing was collected and analyzed for pesticide residues. Typically, two hand washes per day were conducted. If cotton glove samples were used as hand dosimeters, cotton gloves were usually worn under nitrile gloves and should represent the skin. At the end of the day, the cotton gloves were sampled and analyzed for pesticide residues. Data from low crop tractor/vehicle mounted (LCTM) spray applications including 11 independent operator exposure studies (LCTM1-11) are reported in Table 1. Typically, LCTM refers to a broadcast sprayer, where the diluted plant protection product is applied downwards using a large boom. LCTM spray applications are often used in field and row crops. One study (LCTM6) refers to a herbicide application performed in grape vine orchards. The equipment used for this type of application is very different to a field crop sprayer and also application conditions are different to a low crop field situation. Thus, results of this study were not considered for the current evaluation since they were not representative of large-scale spray application using ground boom spray equipment. Five studies determined actual hand exposure by hand washes (LCTM1, 4, 5, 7, and 10). In the remaining studies, cotton gloves were used as surrogates for the determination of actual hand exposure (LCTM2, 3, 8, 9, and 11). Regarding high crop spraying using tractor mounted spray equipment (HCTM), data from eight independent operator exposure studies were reported (HCTM1–8) (Table 1). HCTM typically refers to airblast sprayers that apply diluted plant protection products upwards by creating wind to treat orchard and vineyard crops. Five studies determined actual hand exposure *via* hand washes (HCTM1, 3, 5, 7 and 8) and three studies determined actual hand exposure using cotton gloves as a surrogate (HCTM2, 4 and 6).

**Table 1:** Summary of data from the spray applications performed in low (LCTM) and high (HCTM) crops.

**Study ID**	**Formulation type**	**Application rate (kg a.s.^a^/ha)**	**Water rate (L/ha)**	**Area treated (ha/day)**	**Amount a.s. handled (kg/day)**	
					**M/L^d^**	**Appl.^e^**
**Low crop tractor mounted application (LCTM)**						
Studies where hand exposure was determined by **hand washes**						
LCTM1^b^	Solid	0.5–0.8	100–370	36.0–56.5	21.3–33.0	21.3–33.0
LCTM4	Liquid	0.06	150	50	2.3–3.1	2.3–3.1
LCTM5	Liquid	2.0–4.0	100–250	47–80	160–250	160–250
*LCTM6*	*Solid*	*0.24*	*200*	*3*–*8*	*0.9*–*1.2*	*0.9*–*1.2*
LCTM7	liquid	0.2–0.3	155–238	19–67	4.0–14.0	4.0–14.0
LCTM10	liquid	0.2	200–300	23–180	4.6–31.3	4.6–31.3
	**Minimum:**	**0.2**	**100**	**23**	**2.3**	**2.3**
	**Maximum:**	**4**	**300**	**180**	**250**	**250**
Studies where hand exposure was determined *via* **cotton gloves**						
LCTM2	Liquid	0.25	75–300	*15.2*–*41.2*	3.8–10.3	3.8–10.3
LCTM3	Liquid	1.5	200	5	4.5–8.0	-
LCTM8	Liquid	1.1	100–200	40–60	45.9–68.0	45.9–68.0
LCTM9	Liquid	0.8–1.2	100–200	23–63	25.0–56.5	25.0–56.5
LCTM11	Liquid	4.0	115–400	Ca. 50	157–207	157–207
	**Minimum:**	**0.25**	**75**	**5**	**3.8**	**3.8**
	**Maximum:**	**4**	**400**	**60**	**157.2**	**207**
**High crop tractor mounted application (HCTM)**						
Studies where hand exposure was determined by **hand washes**						
HCTM1^c^	Liquid	0.375	388–1,947	2.9–5.6	1.0–1.4	1.0–1.4
HCTM3	Liquid	0.45	300	5.2–8.4	2.4–3.8	2.4–3.8
HCTM5	Solid	1.4–2.1	300–900	3.0–14.6	7.0–37.8	5.3–30.7
HCTM7	Solid	0.5–1.2	150–640	6.5–12	3.8–13.5	3.8–13.5
HCTM8	Solid	0.06–0.13	80–367	5.3–20	0.8–2.2	0.8–2.2
	**Minimum:**	**0.06**	**80**	**2.9**	**0.8**	**0.8**
	**Maximum:**	**2.1**	**1,947**	**20**	**37.8**	**30.7**
Studies where hand exposure was determined *via* **cotton gloves**						
HCTM2	Liquid	0.6–1.0	200	6–12	3.5–9.1	3.5–9.1
HCTM4	Liquid	0.2	200–230	5.3–11.4	1.1–2.3	1.1–2.3
HCTM6	Liquid	0.4–0.8	400–1,200	5–17	2.4–10.0	2.4–10.0
	**Minimum:**	**0.2**	**200**	**5**	**1.1**	**1.1**
	**Maximum:**	**1.0**	**1,200**	**17**	**10**	**10**

Data from 11 independent operator exposure studies (LCTM1-11) and data from eight independent operator exposure studies (HCTM1-8) are shown. Results from the study highlighted in cursive were not considered in the analysis. For more details, please refer to the text. Minimum and Maximum values are highlighted in bold.

a a.s., active substance;

b LCTM, low crop tractor mounted;

c high crop tractor mounted;

d mixing and loading;

e application.

The AOEM project data were categorized according to LCTM and HCTM and with respect to mixing and loading and application (the number of replicates considered are shown in Supplementary Table 1). With regards to mixing and loading, only data available for liquid formulations were used for the current evaluation since there were no data available for solid formulations using cotton gloves as dosimeter from these studies. Therefore, for each category, i.e., LCTM-mixing and loading, LCTM-application, HCTM-mixing and loading, HCTM-application, it was assumed that the general exposure conditions under which the data were determined are reasonably similar. Accordingly, differences in the results using hand washes vs. the use of cotton gloves are not attributable to different exposure conditions.

For the calculation of normalized exposure values (μg exposure to the active substance/kg active substance used) the respective amount of active substance handled as given in the AOEM report was considered (Supplementary Table 2).

## 3. Results

### 3.1. Actual hand exposure

**Figure 1:**
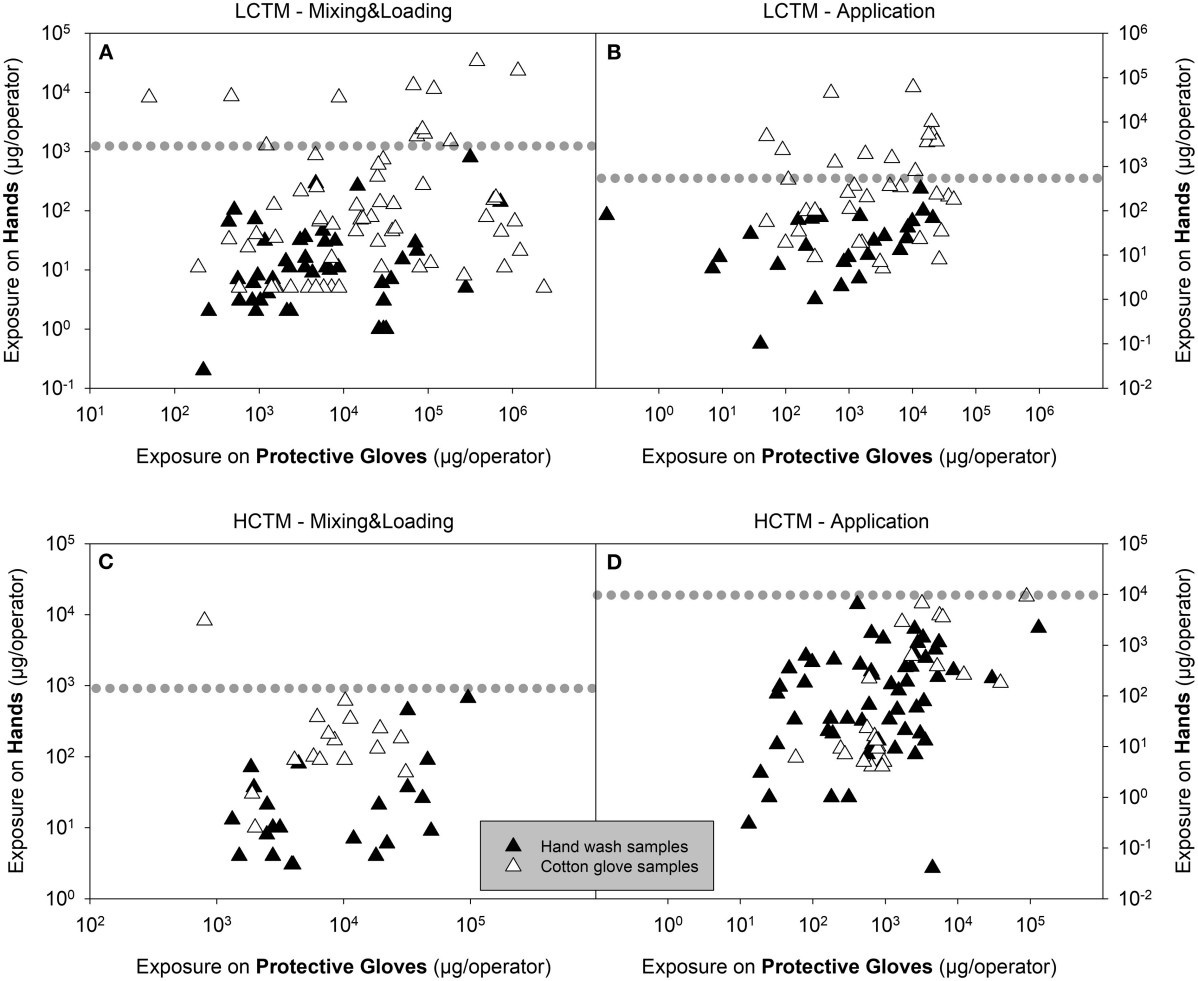
Exposure on hand plotted against exposure on cotton gloves for LCTM **(A,B)** and HCTM **(C,D)** during mixing and loading [M&L, **(A,C)**] or application **(B,D)** of liquid formulation. Black and white triangles represent hand wash samples and cotton glove samples, respectively. The dotted line indicates the level of the highest hand wash sample. Values above the dotted line are from cotton glove samples only.

An overview of the actual exposure, expressed as μg/operator with respect to minimum, maximum and mean values, as well as the 75th and 95th percentile values, are presented in Table 2. Corresponding statistics of the normalized exposure values (μg a.s./kg a.s. handled) are presented in Supplementary Table 2. For LCTM studies, it is notable that above a certain exposure threshold only hand exposure figures determined by cotton gloves can be found for mixing and loading and application (values above the dotted line in Figures 1A,B). This visual effect is not present for the HCTM data.

**Table 2:** An overview of results expressed as μg a.s./operator.

**Parameter**	**Application**							
	**LCTM^a^**				**HCTM^b^**			
	**HW^c^**		**CG^d^**		**HW**		**CG**	
	**Hands^f^**	**P-gloves^e^**	**Hands**	**P-gloves**	**Hands**	**P-gloves**	**Hands**	**P-gloves**
No. of Studies	5	5	4	3	5	5	4	3
No. of replicates	27	18	50	26	61	55	50	26
	[μg a.s./operator]		[μg a.s./operator]		[μg a.s./operator]		[μg a.s./operator]	
Minimum	0.1	0.2	5.0	50.0	0.043	13.0	4.0	58.0
Maximum	819.0	20,866.0	60,496.0	37,000.0	6,765.0	1,30,142.0	66,920.0	88,900.0
Mean	63.9	4,816.3	3,212.1	7,790.6	629.3	4,405.4	3,423.0	7,808.2
75th percentile^*^	44.0	8,325.3	1,527.8	15,516.5	560.0	2,640.0	1,172.5	4,700.0
95th percentile^*^	249.1	15,644.5	7,872.4	23,233.5	2,170.0	6,450.9	9,573.0	37,465.0
	**Mixing and loading (liquids)**							
	**HW**		**CG**		**HW**		**CG**	
	**Hands**	**P-gloves**	**Hands**	**P-gloves**	**Hands**	**P-gloves**	**Hands**	**P-gloves**
No. of studies	4	4	5	4	2	2	2	2
No. of replicates	27	28	61	49	22	22	32	31
	[μg a.s./operator]		[μg a.s./operator]		[μg a.s./operator]		[μg a.s./operator]	
Minimum	1.0	560.0	5.0	50.0	3.0	1,320.0	10.0	800.0
Maximum	802.0	7,17,756.0	33,747.0	11,60,195.0	670.0	96,000.0	10,590.0	30,900.0
Mean	54.0	62,613.5	1,993.8	49,355.3	72.0	18,208.6	721.4	10,768.8
75th percentile^*^	18.5	32,986.8	375.0	27,500.0	37.0	29,500.0	172.50	13,100.0
95th percentile^*^	227.8	3,02,087.3	11,486.0	1,04,302.0	432.0	48,850.0	4,039.0	28,950.0

* Calculated using excel (QUANTIL.INKL; 0.75 for 75th percentile and 0.95 for 95th).

a LCTM, low crop tractor mounted;

b HCTM, high crop tractor mounted,

c HW, hand washes;

d CG, cotton gloves;

e P-Gloves, exposure on protective nitrile gloves;

f hands, hand exposure.

### 3.2. Comparison of actual exposure in LCTM studies

**Figure 2:**
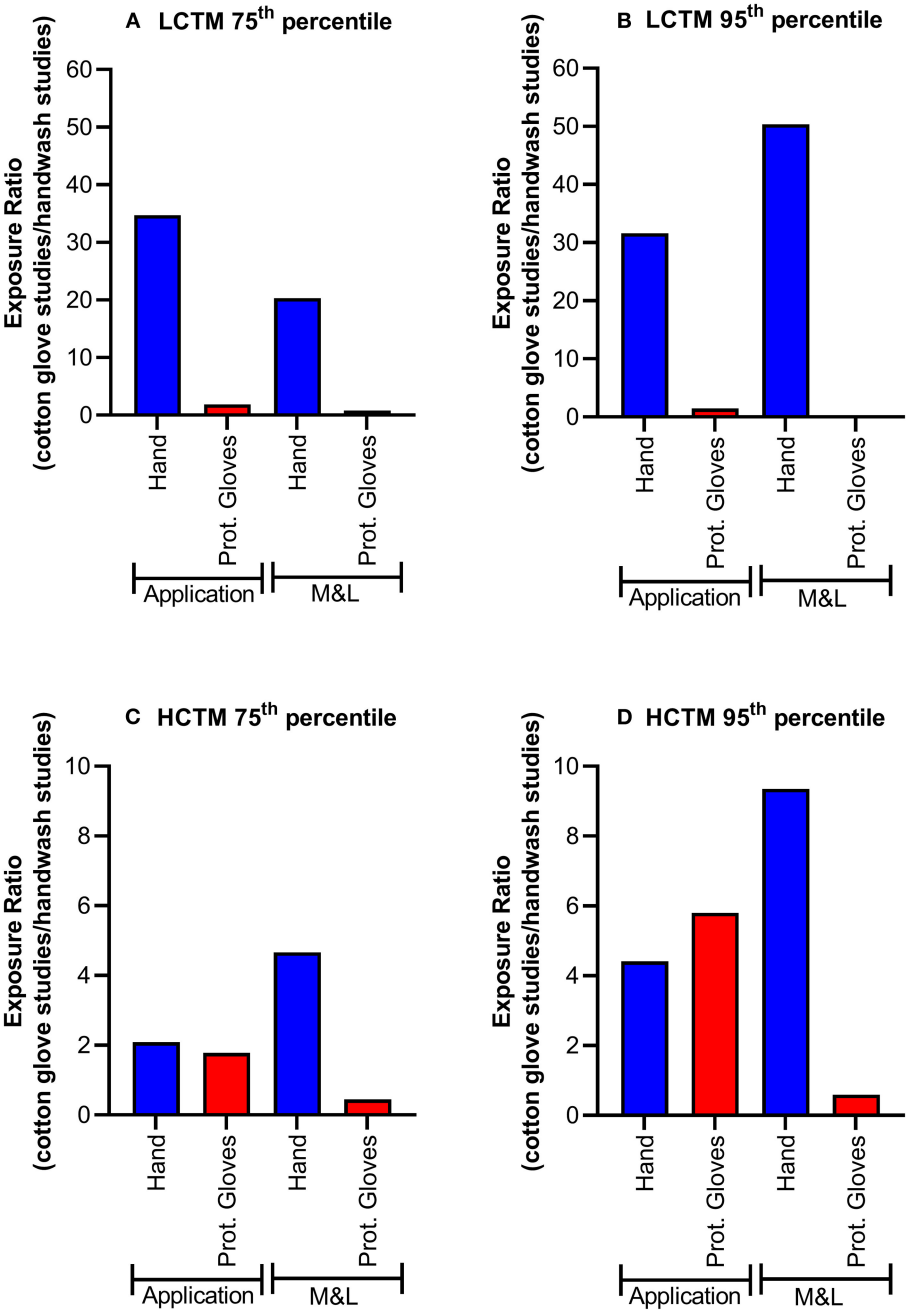
Actual exposure 75th **(A,C)** and 95th percentiles **(B,D)** for LCTM **(A,B)** and HCTM **(C,D)** during mixing and loading (M&L) or application of liquid formulation. A value >1 indicates exposure is higher in cotton gloves studies. Protective gloves (Prot. Gloves) were used as a reference.

Exposure results (μg a.s./operator) for actual hand exposure determined by the hand wash technique and *via* cotton gloves as well as exposures determined on protective gloves are summarized in Table 2. The actual hand exposure during application determined *via* hand washes covered the range of 0.1–819 μg a.s./operator, with a mean value of 63.9 μg a.s./operator. By contrast, when cotton gloves were used as the dosimeter (surrogate skin) to determine actual hand exposure during application, the range was from 5 to 60,469 μg a.s./operator, with the mean of 3,212.1 μg a.s./operator. The 75th and 95th percentile values for hand exposure determined *via* cotton glove sampling were 34.7- and 31.6-fold higher than when determined *via* hand washes, respectively (Figures 2A,B). There was less than a 2-fold difference in the 75th and 95th percentile exposure values determined using protective gloves after hand washing or cotton gloves. The values were consistently higher in the studies using cotton gloves as the dosimeter to determine actual hand exposure. Comparable results were obtained when comparing normalized exposure values.

Although the factor of difference varied, similar results were obtained for the mixing and loading of liquid formulations (Figures 2A,B). Again, hand exposure determined by hand washes were lower than those determined by cotton gloves: the 75th and 95th percentile values for hand exposure determined *via* cotton glove sampling were 20.3- and 50.4-fold higher than when determined *via* hand washes, respectively, whereas here as well 75th and 95th percentile values regarding protective gloves were in a much more comparable range (1.2- and 2.9-fold difference, respectively). For application in High-Crop Tractor Mounted (HCTM), exposure ratios for hand and protective gloves were in a similar range (Figures 2C,D).

### 3.3. Comparison of actual exposure in HCTM studies

As for LCTM, actual hand exposure determined using hand washes were always lower compared the results using cotton gloves when considering 75th and 95th percentile values (Table 2). Regarding application the 75th and 95th percentile values for hand exposure determined *via* cotton glove sampling were 2.1- and 4.4-fold higher than when determined *via* hand washes, respectively (Figures 1C,D). In this case approximately similar factors apply for the exposures determined on protective gloves (Figures 1C,D).

For mixing and loading the 75th and 95th percentile values for hand exposure determined *via* cotton glove sampling were 4.7- and 9.3-fold higher than when determined *via* hand washes, respectively (Figures 1C,D) whereas considering 75th and 95th percentile values exposures determined on protective gloves were even lower in the hand wash studies (Table 2 and Figures 1C,D).

## 4. Discussion

Measurement of the dermal exposure to PPPs during the various activities of agricultural workers is a critical aspect of their risk assessment. While conservative methods to determine exposure are protective, they may be unrealistic, which could also lead to misleading conclusions. For example, when personal protection equipment recommendations are driven by the conservatism of the exposure measurement. Conservative methods can also be misleading when evaluating the extent of protection of protective clothing and gloves.

The method by which dermal exposure is measured has changed over the past few decades. A wipe method was commonly used until the early 1990s (10); however, this method was not standardized at the time and only provides information about the mass of contaminant on a surface, and does not relate the mass of contaminant on a surface to the mass transferred to a worker's skin (10). A study in 1999 by Fenske et al. (11) made a direct comparison of glove, handwash and wipe methods to measure hand exposure of apple thinners to a pesticide (Guthion). They showed that the glove method resulted in a 2.4-fold overestimate of exposure, whereas the wipe method produced a 10-fold underestimate. They also recommended that studies measuring hand exposure to pesticides should include a careful description of sampling methods, as well as recognize the potential for bias in the measurements. Another study based on controlled experiments compared cotton gloves and cotton wipe sampling methods for exposure to liquids, and cotton wipe and hand rinsing methods for exposure to powders (8). They showed that wipe and rinse methods produced comparable exposure values (at least, within 5-fold), whereas values using cotton gloves were as high as 42-fold higher than using the wipe method, with the ratio being higher at lower levels of exposure. Despite the reported higher measured exposures using cotton gloves, these continue to be a main method of sampling. For example, a recent review based on non-controlled field studies of exposure of workers in apple growing in France noted that hand exposure was mainly measured using absorbent gloves, whereas hand rinsing methods were rarely used (12). These studies highlighted the importance of standardizing and validating methods for dermal exposure assessment to be able to compare more accurate estimates of pesticide exposure (11). The current evaluation is based on AOEM data from 30 experiments carried out under standardized conditions in test chambers, following detailed protocols to increase reproducibility and thus represents a robust dataset with which to compare hand wash and cotton glove measurements of exposure. This evaluation indicates that actual hand exposure determined using cotton gloves resulted in higher exposure values compared to those determined using hand washes. This finding is in accordance with Fenske et al. and others who have demonstrated that cotton glove dosimeters consistently estimate much higher exposure levels relative to hand washes (8, 13, 14). Given that related exposures determined on protective gloves were in almost similar ranges, indicating similar exposure conditions, this finding cannot be attributed to different exposure conditions. One explanation to account for the higher exposures determined using cotton gloves is the higher retention of the compound in the cotton gloves compared to human skin. This was also noted by others who suggested that cotton gloves (as well as cotton patches) may act as reservoirs for the substances and quickly become saturated (15, 16). Cotton gloves are also likely to rapidly absorb liquids, thus resulting in an overestimation of exposure (8). Another possibility is the lower removal efficiency of the PPP from the hands during washing due to potential dermal uptake (4).

To consider the impact of dermal uptake on hands during an operator exposure study, hand wash data can be adjusted by using product-specific dermal absorption values. The use of dermal absorption data from human *in vitro* skin studies following OECD test guideline No. 428 (17) is currently accepted for higher tier risk assessments in Europe. The outcome of the study could be either evaluated according to the EFSA guidance on dermal absorption (18), or by following new approaches where the surface area exposed and the duration of exposure are more influential than the load (19).

In the absence of any experimentally derived dermal absorption data, hand wash data could also be adjusted using default dermal absorption values proposed by EFSA (10) or according to Aggarwal et al. (20) for concentrates.

To consider the impact of dermal uptake on hands during an operator exposure study, hand wash exposure data could be adjusted (21) by using product-specific dermal absorption values. The use of dermal absorption data from human *in vitro* skin studies following OECD test guideline No. 428 (17) is currently accepted for higher tier risk assessments in Europe. The outcome of the study could be either evaluated according to the to the local regulatory guidance, e.g. EFSA guidance on dermal absorption (18), holistic, data driven assessments (22) or by following new approaches where the surface area exposed, and the duration of exposure are more influential than the load.

In the absence of any experimentally derived dermal absorption data, hand wash data could also be adjusted using default dermal absorption values proposed by EFSA (10) or according to Aggarwal et al. According to EFSA, for concentrates, a default dermal absorption value of 10% is used for solids and water based liquid concentrates. A default dermal absorption value of 25% is used for organic solvent based liquid concentrates. For the respective dilutions, the default values of 50 and 70% are proposed. Based on these values the following adjustment factors were derived to account for a potential dermal uptake:

Water based concentrates/solids:

- Concentrate (default 10% dermal absorption): 1.1 (=1/0.90)
- Dilution (default 50% dermal absorption): 2.0 (=1/0.50)

Organic solvent based:

- Concentrate (default 25% dermal absorption): 1.3 (=1/0.75)
- Dilution (default 70% dermal absorption): 3.3 (=1/0.3)

In general, the adjustment factors established based on a default approach are still far lower than the differences observed between hand wash data and cotton glove data. For future exposure studies, where hand wash samples are used to determine hand exposure, the conduct of a parallel dermal absorption assay with the same product might be useful to better define the dermal absorption adjustment factor. The potential dermal uptake accounts for only a small portion of the difference observed between the hand wash and cotton glove data. These findings strongly support the conclusion that a substantially higher retention capacity of cotton gloves is the main parameter leading to the markedly higher actual exposures determined with cotton gloves.

## 5. Conclusion

This evaluation of the AOEM data indicates that the cotton glove method results in much higher levels of measured hand exposure than the hand wash method. It cannot be excluded that dermal uptake could have contributed to that result; however, the findings suggest the higher retention capacity of cotton gloves vs. human skin to be the main impact parameter. The cotton glove method does not provide the results with regards to the protection level that can be expected from the use of protective gloves. Therefore, we believe that the application of the hand wash method is a more accurate measure of exposure levels, if either specific dermal absorption data or, in its absence, default assumptions are applied as adjustment factor.

## Data availability statement

The original contributions presented in the study are included in the article/Supplementary material, further inquiries can be directed to the corresponding author.

## Author contributions

CK, NH, and GH were substantial contributors to underlying work. NH drafted the manuscript. CK and GH edited the manuscript. All authors approved the final version and agree to take responsibility for the work.
