# Supplementary material for: Dermal measurement of exposure to plant protection products: Actual hand exposure from hand washing vs. wearing cotton gloves
Source: Front Public Health. 2022 Dec 15;10:1037780. doi: 10.3389/fpubh.2022.1037780 (PMC9798894; doi:10.3389/fpubh.2022.1037780)
Supplement: Supplementary file 1 [file Table_1.docx]

Supplementary Material

**Supplementary Table 1.** Categorization of studies according to LCTM and HCTM and with respect to “mixing and loading” and “application”.

| **Study conditions** | **Study ID** | **Number of replicates^1)^** | | | | | | | |
| --- | --- | --- | --- | --- | --- | --- | --- | --- | --- |
|  |  | **Mixing and loading liquids** | | | | **Application** | | | |
|  |  | **Hand exposure determined via hand wash** | | **Hand exposure determined via cotton gloves** | | **Hand exposure determined via hand wash** | | **Hand exposure determined via cotton gloves** | |
|  |  | **hands** | **P-gloves** | **hands** | **P-gloves^1)^** | **hands** | **P-gloves** | **hands** | **P-gloves** |
| LCTM | LCTM1 | *7 (WG)^2)^* | 7 (WG)^2)^ | - |  | 7 | 5 |  |  |
|  | LCTM2 |  |  | 13 (7) | 15 |  |  | 13 (3) | 7 (2) |
|  | LCTM3 |  |  | 10 | 10 |  |  | Not sampled | Not sampled |
|  | LCTM4 | 3 | 4 |  |  | 3 | 1 |  |  |
|  | LCTM5 | 12 | 12 |  |  | 12 | 7 |  |  |
|  | LCTM7 | 5 | 5 |  |  | 2 | 2 |  |  |
|  | LCTM8 |  |  | 10 | 10 |  |  | 9 | 9 |
|  | LCTM9 |  |  | 16 (3) | 16 (1) |  |  | 16 (1) | 10 (1) |
|  | LCTM10 | 7 | 7 |  |  | 3 | 3 |  |  |
|  | LCTM11 |  |  | 12 | *12^3)^* |  |  | 12 | *12^3)^* |
|  | **Total:** | **27^4)^** | **28** | **61 (10)^5)^** | **51 (1)** | **27^6)^** | **18** | **50 (4)^7)^** | **26 (2)^8)^** |
|  |  |  |  |  |  |  |  |  |  |
| HCTM | HCTM1 | 10 | 10 |  |  | 10 | 10 |  |  |
|  | HCTM2 |  |  | 15 (15) | *15^3^* |  |  | 15 (15) | *15 (1)^3)^* |
|  | HCTM3 | 12 | 12 |  |  | 12 | 12 |  |  |
|  | HCTM4 |  |  | Not sampled | Not sampled |  |  | 12 | 12 |
|  | HCTM5 | 12 (WG)*^2)^* | 12 (WG)*^2)^* |  |  | 15 | 10 |  |  |
|  | HCTM6 |  |  | 17 | 16 |  |  | 17 | 10 |
|  | HCTM7 | *12 (WG)^2)^* | *12 (WG)^2)^* |  |  | 8 | 8 |  |  |
|  | HCTM8 | Not sampled | Not sampled |  |  | 16 | 15 |  |  |
|  | **Total:** | **22** | **22** | **32 (15)** | **16** | **61^9)^** | **55** | **44 (15)** | **22** |
| 1): Values in brackets refer to number of replicates for which the report indicates that the measured exposure was below the Limit of quantification (LOQ) and ½ LOQ was taken into account.  2): Data not considered for the evaluation as no cotton glove data are available for WG formulation  3): Exposure on protective gloves was determined via cotton gloves worn above protective gloves, therefore data are not considered.  4): One additional data point was reported as non detects and therefore not considered for the evaluation.  5): Two additional data point were reported as non detects and therefore are not considered for the evaluation.  6): Eight additional data point were reported as non detects and therefore not considered for the evaluation.  7): One additional data point was reported as non detect and for a further value it is indicated that the value was not used for the AOEM model. Therefore, the data were not considered for the evaluation.  8): Four additional data point were reported as non detects and therefore not considered for the evaluation.  9): Four additional data point were reported as non detects and therefore not considered for the evaluation. | | | | | | | | | |

**Supplementary Table 2: An overview of results expressed as normalized exposure values (µg a.s./kg a.s. handled)**.

| **Parameter** | **Application** | | | | | | | |
| --- | --- | --- | --- | --- | --- | --- | --- | --- |
|  | **LCTM** | | | | **HCTM** | | | |
|  | **HW** | | **CG** | | **HW** | | **CG** | |
|  | **hands** | **P-gloves** | **hands** | **P-gloves** | **hands** | **P-gloves** | **hands** | **P-gloves** |
| No. of Studies: | 5 | 5 | 4 | 3 | 5 | 5 | 4 | 3 |
| No. of replicates: | 27 | 18 | 50 | 26 | 61 | 55 | 50 | 26 |
|  | [µg a.s./kg a.s.] | | [µg a.s./kg a.s.] | | [µg a.s./kg a.s.] | | [µg a.s./kg a.s.] | |
| Minimum: | 0.0 | 0.0 | 0.0 | 1.1 | 0.0 | 1.0 | 2.2 | 32.2 |
| Maximum: | 4.14 | 359.5 | 1063.8 | 960.0 | 3303.3 | 7436.7 | 18588.9 | 24694.4 |
| Mean: | 1.03 | 71.3 | 67.9 | 198.6 | 235.9 | 889.1 | 797.6 | 1920.1 |
| 75^th^ percentile: | 1.77 | 71.0 | 45.0 | 290.8 | 181.9 | 1141.9 | 208.4 | 956.0 |
| 95^th^ percentile: | 3.72 | 353.1 | 229.0 | 704.3 | 1435.8 | 2880.0 | 2595.4 | 5951.6 |
|  | **Mixing & Loading (liquids)** | | | | | | | |
|  | **HW** | | **CG** | | **HW** | | **CG** | |
|  | **hands** | **P-gloves** | **hands** | **P-gloves** | **hands** | **P-gloves** | **hands** | **P-gloves** |
| No. of Studies: | 4 | 4 | 5 | 4 | 2 | 2 | 2 | 2 |
| No. of replicates: | 27 | 28 | 61 | 49 | 22 | 22 | 32 | 31 |
|  | [µg a.s./kg a.s.] | | [µg a.s./kg a.s.] | | [µg a.s./kg a.s.] | | [µg a.s./kg a.s.] | |
| Minimum: | 0.0 | 46.7 | 0.0 | 2.0 | 0.8 | 500.0 | 2.0 | 111.1 |
| Maximum: | 18.93 | 2871.0 | 595.2 | 25276.6 | 186.1 | 26666.7 | 2786.8 | 5138.9 |
| Mean: | 1.66 | 657.2 | 46.7 | 1640.0 | 28.4 | 6250.6 | 148.0 | 2116.4 |
| 75^th^ percentile: | 1.69 | 919.5 | 17.1 | 1564.4 | 24.0 | 8500.0 | 37.6 | 3094.2 |
| 95^th^ percentile: | 4.18 | 1961.5 | 259.2 | 5846.9 | 161.7 | 17390.6 | 566.3 | 14393.0 |
